# Diagnostic Performance of GPT-4o and Claude 3 Opus in Determining Causes of Death From Medical Histories and Postmortem CT Findings

**DOI:** 10.7759/cureus.67306

**Published:** 2024-08-20

**Authors:** Masanori Ishida, Wataru Gonoi, Keisuke Nyunoya, Hiroyuki Abe, Go Shirota, Naomasa Okimoto, Kotaro Fujimoto, Mariko Kurokawa, Motoki Nakai, Kazuhiro Saito, Tetsuo Ushiku, Osamu Abe

**Affiliations:** 1 Department of Radiology, Tokyo Medical University Hospital, Tokyo, JPN; 2 Department of Radiology, The University of Tokyo Hospital, Tokyo, JPN; 3 Department of Pathology, The University of Tokyo Hospital, Tokyo, JPN

**Keywords:** cause of death, postmortem computed tomography, large language model, gpt-4o, claude 3 opus, artificial intelligence

## Abstract

Introduction: This study evaluates the diagnostic performance of the latest large language models (LLMs), GPT-4o (OpenAI, San Francisco, CA, USA) and Claude 3 Opus (Anthropic, San Francisco, CA, USA), in determining causes of death from medical histories and postmortem CT findings.

Methods: We included 100 adult cases whose postmortem CT scans were diagnosable for the causes of death using the gold standard of autopsy results. Their medical histories and postmortem CT findings were compiled, and clinical and imaging diagnoses of both the underlying and immediate causes of death, as well as their personal information, were carefully separated from the database to be shown to the LLMs. Both GPT-4o and Claude 3 Opus generated the top three differential diagnoses for each of the underlying or immediate causes of death based on the following three prompts: 1) medical history only; 2) postmortem CT findings only; and 3) both medical history and postmortem CT findings. The diagnostic performance of the LLMs was compared using McNemar’s test.

Results: For the underlying cause of death, GPT-4o achieved primary diagnostic accuracy rates of 78%, 72%, and 78%, while Claude 3 Opus achieved 72%, 56%, and 75% for prompts 1, 2, and 3, respectively. Including any of the top three differential diagnoses, GPT-4o’s accuracy rates were 92%, 90%, and 92%, while Claude 3 Opus’s rates were 93%, 69%, and 93% for prompts 1, 2, and 3, respectively. For the immediate cause of death, GPT-4o’s primary diagnostic accuracy rates were 55%, 58%, and 62%, while Claude 3 Opus’s rates were 60%, 62%, and 63% for prompts 1,2, and 3, respectively. For any of the top three differential diagnoses, GPT-4o’s accuracy rates were 88% for prompt 1 and 91% for prompts 2 and 3, whereas Claude 3 Opus’s rates were 92% for all three prompts. Significant differences between the models were observed for prompt two in diagnosing the underlying cause of death (p = 0.03 and <0.01 for the primary and top three differential diagnoses, respectively).

Conclusion: Both GPT-4o and Claude 3 Opus demonstrated relatively high performance in diagnosing both the underlying and immediate causes of death using medical histories and postmortem CT findings.

## Introduction

Large language models (LLMs) are neural network models trained on vast amounts of text data, demonstrating their high performance in natural language processing tasks [1]. They can be utilized across various fields, including medicine [2]. Regarding the diagnostic capabilities of LLMs in radiology, it has been reported that these models can provide a list of highly probable differential diagnoses and their respective accuracies based solely on textual information from clinical histories and imaging findings [3-5]. Thus, the potential use of language artificial intelligence (AI) in diagnostic imaging has been revealed. Recently, various manufacturers have developed their own LLMs, which have undergone multiple version upgrades. In the area of death investigation, the diagnostic abilities of AI models using radiological images have not yet been investigated. With the shortage of forensic doctors, pathologists, and radiologists specializing in death investigation, the clinical implementation of AI-based diagnostic aids is expected, and studies of deep learning models using postmortem CT images have been reported [6]. Therefore, in this study, we employed the latest versions of GPT-4o (OpenAI, San Francisco, CA, USA) [7] and Claude 3 Opus (Anthropic, San Francisco, CA, USA) [8] to assess their diagnostic performance in determining the cause of death using medical histories and imaging findings.

## Materials and methods

This case-control study was approved by the Institutional Review Board of the University of Tokyo Hospital (approval no. 2076). Written informed consent was obtained from the families of the deceased. All procedures conformed to the ethical principles outlined in the Declaration of Helsinki. In this study, we included 100 cases of adult in-hospital natural deaths that occurred between May 2014 and June 2019, whose postmortem CT was diagnosable for the causes of death with the gold standard of autopsy results. 

The compiled medical histories and postmortem CT findings of all adult cases were blinded to the autopsy results. Postmortem CT reports were written independently by two board-certified diagnostic radiologists at our tertiary referral center. In the interpretation, all organs and structures of the postmortem CT scan (the average time elapsed from death to postmortem CT is six hours and 40 minutes) from the head to the lower extremities were systematically evaluated. When antemortem CT images were available, they were referred to while interpreting the postmortem CT images and writing the report. Postmortem CT reports written in Japanese were double-checked and finalized by consensus, then collected and used for input. Medical histories were prepared by the attending physician for postmortem CT and pathological autopsy. The medical histories were prepared in a manner similar to writing discharge summaries. Medical histories include present illness at admission, past history (pre-existing or pathologically diagnosed diseases), social life history (including tobacco use, alcohol consumption, occupation, etc.), and family history. Detailed hematological, pathological, biochemical, and radiological reports were not included in the medical histories.

**Figure 1:**
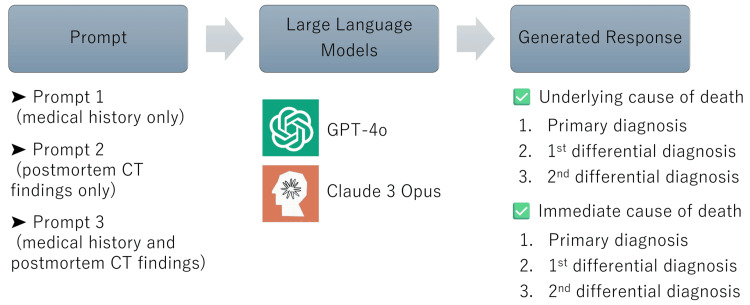
Overview of the study

**Figure 2:**
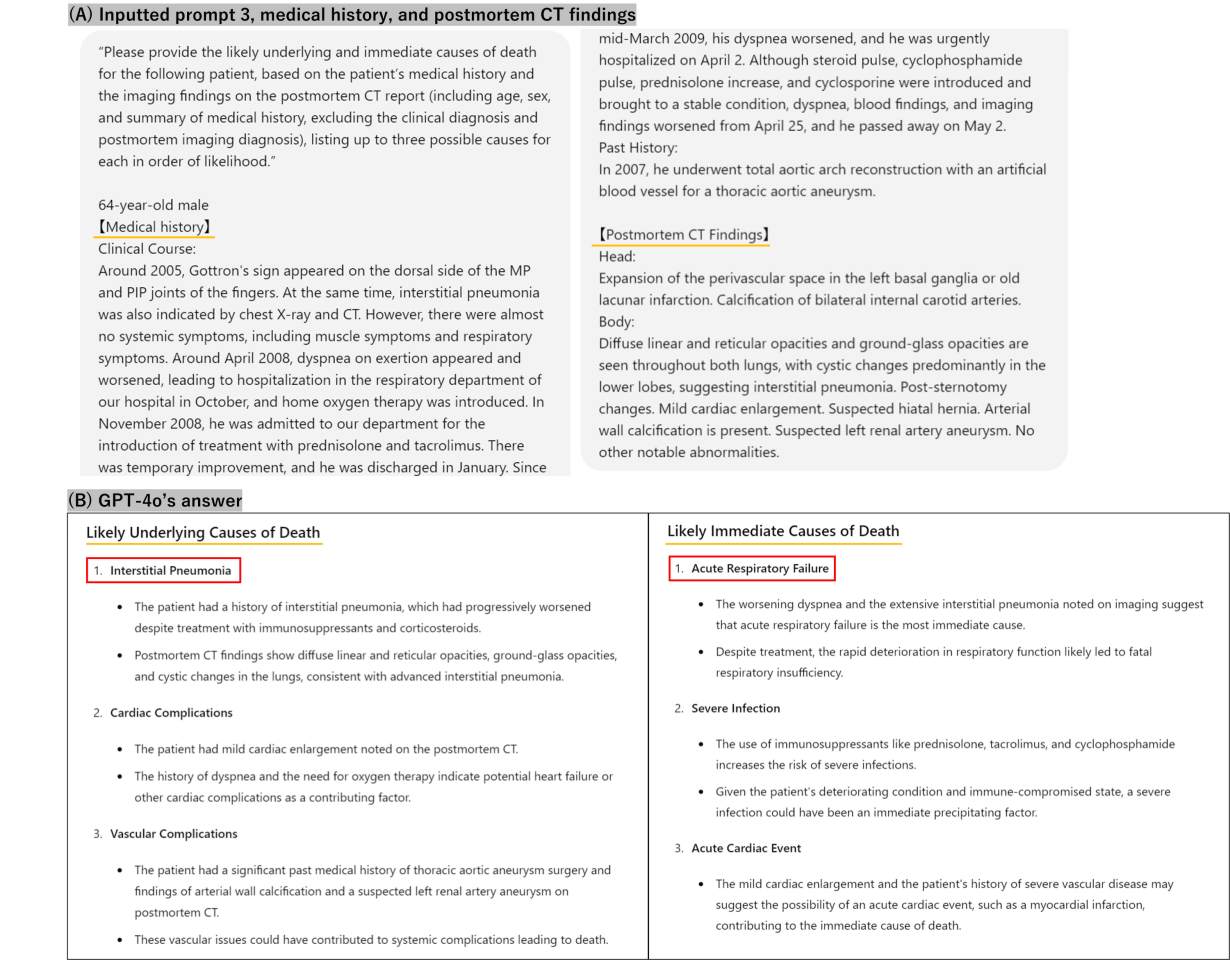
Question for prompt 3 and the response samples (A) Manually typed question for prompt three, medical history, and postmortem CT findings; (B) Response generated by GPT-4o The language used in the study is Japanese, but this example is shown in English. In this case, the underlying cause of death was interstitial pneumonia, and the immediate cause of death was respiratory failure, consistent with diagnoses from autopsy and CT.

An overview of this study is presented in Figure 1. We used GPT-4o (OpenAI), released on May 13, 2024, and Claude 3 Opus (Anthropic), released on March 4, 2024, to list a primary diagnosis and two differential diagnoses of the underlying and immediate cause of death, respectively. When showing the data (text only) to the LLMs, medical and imaging diagnoses of the underlying and immediate causes of death, as well as personal information, were carefully separated from the database. In defining causes of death, the following two distinct levels can be distinguished: the underlying cause of death (the primary underlying disease that initiates the events leading to death) and the immediate cause of death (the final disease or condition resulting in death) [9,10]. Because all medical histories and postmortem CT reports were in Japanese, textual data without a fixed format was passed to the models without translation. The LLMs were asked to present two differential diagnoses of the cause of death in addition to the primary diagnosis in order of likelihood (Figure 2).

The three prompts

Analyses were conducted under the following three prompts after inputting the definitions of the underlying and immediate causes of death above. Prompt 1: “Please provide the likely underlying and immediate causes of death for the following patient, based on the patient’s medical history (including age, sex, and summary of medical history, excluding the clinical diagnosis), listing up to three possible causes for each in order of increasing likelihood.” Prompt 2: “Please provide the likely underlying and immediate causes of death for the following patient, based on the imaging findings on the postmortem CT report (including age and sex but excluding the summary of the patient’s medical history, clinical diagnosis, and postmortem imaging diagnosis), listing up to three possible causes for each in order of likelihood.” Prompt 3: “Please provide the likely underlying and immediate causes of death for the following patient, based on the patient’s medical history and the imaging findings on the postmortem CT report (including age, sex, and summary of medical history, excluding the clinical diagnosis and postmortem imaging diagnosis), listing up to three possible causes for each in order of likelihood.”

Each condition was examined using a separate thread in an independent session to prevent previous inputs from influencing subsequent ones. The underlying and immediate causes of death generated by LLMs were determined to be consistent with the postmortem CT and autopsy diagnoses. The accuracy of the LLMs’ primary diagnosis and two differential diagnoses each for the underlying and immediate causes of death were determined by a consensus of two board-certified diagnostic radiologists with 17 to 18 years of experience in postmortem imaging. When there was a difference in expression or choice of words between the pathologist’s diagnosis by autopsy and the AI’s diagnosis by LLMs, it was considered accurate if it was determined to be synonymous. Ambiguous answers or those lacking sufficient elements were considered incorrect.

McNemar’s test was used to assess differences in correct response rates for the primary and three major differential diagnoses under prompts 1, 2, and 3. We also examined whether there was a difference between the two LLMs in the number of cases in which the top three differential diagnoses could not be listed by either prompt. Two-sided p-values <0.05 were considered statistically significant. Statistical analyses were performed using SPSS Statistics version 29.0 (IBM Corp., Armonk, NY, USA).

## Results

The classification of diseases for the underlying and immediate causes of death is shown in Table 1 and Table 2, respectively. The diagnostic accuracy of each model is summarized in Table 3. For the underlying cause of death, GPT-4o achieved primary diagnostic accuracy rates of 78%, 72%, and 78% for prompts 1, 2, and 3, respectively. Claude 3 Opus achieved 72%, 56%, and 75%, respectively. When considering any of the top three differential diagnoses, GPT-4o’s accuracy rates were 92%, 90%, and 92%, while Claude 3 Opus’s rates were 93%, 69%, and 93% for prompts 1, 2, and 3, respectively. For the immediate cause of death, GPT-4o’s primary diagnostic accuracy rates were 55%, 58%, and 62%, compared with Claude 3 Opus’s 60%, 62%, and 63% for prompts 1, 2, and 3, respectively. For any of the top three differential diagnoses, GPT-4o’s accuracy rates were 88% for prompt 1 and 91% for prompts 2 and 3, whereas Claude 3 Opus’s were 92% for all three prompts.

**Table 1:** Underlying causes of death by type of pathology

Type of pathology	No. of cases (n = 100)
Solid malignancy	43
Cardiovascular	27
Hematological malignancy	18
Respiratory disease	5
Autoimmune disease	2
Liver disease	2
Infectious disease	1
Metabolic disorder	1
Neurologic disease	1

**Table 2:** Immediate causes of death by type of pathology

Type of pathology	No. of cases (n = 100)
Respiratory failure	43
Heart failure	16
Multiorgan failure	16
Intracranial hemorrhage	5
Cardiac tamponade	4
Liver failure	4
Circulatory failure	3
Hemorrhagic shock	3
Brain stem metastasis	2
Septic shock	2
Cerebral infarction	1
Hypoxic encephalopathy	1

**Table 3:** Diagnostic accuracy of large language models * A p-value of <0.05 was considered statistically significant.

Causes	Accuracy (%)		McNemar's test (p-value)
	GPT-4o	Claude 3 Opus	GPT-4o vs. Claude 3 Opus
Underlying cause of death			
Primary diagnosis by prompts 1, 2, and 3	78%, 72%, 78%	72%, 56%, 75%	0.21/*0.03/0.549
Top three differential diagnoses by prompts 1, 2, and 3	92%, 90%, 92%	93%, 69%, 93%	1.000/*<0.01/1.000
Immediate cause of death			
Primary diagnosis by prompts 1, 2, and 3	55%, 58%, 62%	60%, 62%, 63%	0.441/0.617/1.000
Top three differential diagnoses by prompts 1, 2, and 3	88%, 91%, 91%	92%, 92%, 92%	0.344/1.000/1.000

McNemar’s test demonstrated significant differences in determining the underlying cause of death between the two models for prompt 2 (p = 0.03 and <0.01 for determining the primary diagnosis and the top three diagnoses, respectively). No significant differences in determining the underlying cause of death between the models were observed for prompts 1 and 3. Additionally, no significant differences in determining the immediate cause of death between the models were observed for prompts 1, 2, and 3. The results are summarized in Table 3. There was also no significant difference between the two LLMs in the number of cases in which the top three differential diagnoses could not be listed by either prompt (p = 0.625 and <0.25 for determining underlying and immediate causes of death, respectively (Table 4)). The underlying causes of death for which GPT-4o was not listed are as follows: two cases of dilated cardiomyopathy, one case of dermatomyositis, one case of liver cirrhosis, one case of hepatocellular carcinoma, and one case of pulmonary tuberculosis. Of these, the same two cases of dilated cardiomyopathy and one case of pulmonary tuberculosis were also not listed by Claude 3 Opus as the underlying cause of death. In addition, one case of esophageal tumor was not listed as an underlying cause of death by Claude 3 Opus. The immediate causes of death for which GPT-4o was not listed are as follows: two cases of multiple organ failure and one case of peritonitis.

**Table 4:** Comparison of the number of cases in which the top three differential diagnoses are not listed by any of the prompts between the two models

	GPT-4o vs. Claude 3 Opus	McNemar's test (p-value)
Underlying cause of death	6/100 vs. 4/100	0.625
Immediate cause of death	3/100 vs. 0/100	0.25

## Discussion

To the best of our knowledge, this is the first study to use generative AI to make cause-of-death diagnoses. In this study, we evaluated the diagnostic performance of the language AI models, GPT-4o and Claude 3 Opus, in determining the cause of death based on three types of input information: medical history only, postmortem CT findings only, and medical history with postmortem CT findings. The results showed that GPT-4o and Claude 3 Opus demonstrated relatively high performance in diagnosing both the underlying and immediate causes of death by inputting postmortem CT findings in addition to the medical histories. It could be said that the usefulness of AI in cause-of-death determination has been demonstrated, potentially reflecting the diagnostic performance of AI models.

In determining the top three differential diagnoses, both underlying and immediate causes of death were estimated with levels of accuracy of around 90% for all prompts, except for the method of diagnosing the underlying cause of death in Claude 3 Opus with prompt 2. It can be said that GPT-4o can diagnose with a high degree of accuracy for differential diagnosis, but Claude 3 Opus cannot achieve the same level.

However, the accuracy of the primary diagnosis of the immediate cause of death for both GPT-4o and Claude 3 Opus is moderate (about 50% to 60%). Among the prompts, the accuracy was higher when both history and postmortem CT findings were inputted and slightly higher when only postmortem CT findings were inputted compared to when only medical history was inputted. This suggests that when a generative AI diagnoses the cause of death, having both history and CT findings is generally more useful than having just one. The additional information provided in this study was also helpful.

The diagnostic accuracy rates of GPT-4o and Claude 3 Opus were similar, except for prompt 2, in diagnosing the underlying cause of death. The difference in prompt 2’s results might be because not all of the relevant medical history is reflected in a report of a single imaging study, and the patient’s clinical course is crucial in understanding the cause of death. For prompt 2, in diagnosing the underlying cause of death, Claude 3 Opus demonstrated significantly inferior diagnostic performance compared with GPT-4o when given only the postmortem CT findings as input, without the textual information of medical histories. It is difficult to determine the reasons for the differences in diagnostic performance between the models observed in this study. In the estimation of underlying and immediate causes of death, prompt 3 for both GPT-4o and Claude 3 Opus had similar or higher diagnostic accuracy than prompts 1 and 2. This suggests that both medical history and postmortem CT findings would be better for cause-of-death determination by LLMs.

Regarding previous studies, there is a paper evaluating the diagnostic performance of GPT-4 and Claude 3 Opus in answering radiology’s diagnosis. The questions are based solely on textual information from clinical histories and imaging findings. With the input of text information from clinical histories and imaging findings, GPT-4, the previous version of GPT-4o, correctly answered 54% of the questions [3]. The accuracy of primary diagnoses obtained by Claude 3 Opus from clinical histories plus imaging findings was 55.3% [11]. These percentages obtained in both studies were similar, suggesting that the abilities of the two models were similar. Additionally, there is a pre-print article comparing the diagnostic performance of GPT-4o and Claude 3 Opus, and the diagnostic accuracy of Claude 3 Opus was superior to that of GPT-4o for reasons that are still unclear [12].

In this study, there was no difference in the number of cases that could not be listed as the top three diagnoses in the two models. There may be strengths and weaknesses in reasoning between the models; however, it is unclear what quirks exist in the models. There was no significant difference in the number of cases in which a differential diagnosis was not made, indicating that the use of either model is currently acceptable.

Although the diagnostic performance of LLMs is high, the basis for their decisions may not coincide with that of medical specialists in a reasonable number of cases. Even LLMs with high diagnostic performance in medical imaging may not have a high degree of concordance between the rationale for the decision and the findings that the specialist emphasized. Furthermore, the information and prompts provided to LLMs were found to be important. This raises concerns about the validity of cause-of-death diagnoses in LLMs, as there is a risk that inappropriate information may lead to unexpected diagnoses in practice.

Nevertheless, this study has some limitations. First, there is a bias in the distribution of included cases. A small number of cases had hemorrhagic lesions (12/100) that could be detected by postmortem CT. It could be considered a pilot study in terms of the limited number of cases and possible bias in cause-of-death groups. More cases are expected to be examined. Second, there is a potential bias arising from the use of non-standardized medical histories and postmortem CT reports and the variability in the information provided by different physicians. The degree to which the medical history is summarized, i.e., whether it is detailed or more concise, varies depending on the physician who treated the patient. While it is undeniable that the prepared histories did not vary from physician to physician and included information essential for postmortem CT interpretation and pathological autopsy as directed, it remains unclear whether a history that does not directly lead to death or one more focused on information related to death is preferable when assessing the cause of death by LLMs, which were diagnosed using information available on the Internet in this study. Third, some cases resulted in differences in cause-of-death representation between the radiologist and the pathologist. We cannot deny the possibility that the same pathological condition may have been assumed but expressed differently when verbalized. Additionally, although the gold standard for diagnosing the cause of death used in this study was a pathology diagnosis by autopsy, it is unlikely that differences in cause-of-death diagnoses among pathologists, including the possibility of multiple coexisting causes of death, do not exist. Fourth, the LLMs' outputs are likely to differ depending on whether the input is in Japanese or English.

When assisting in determining the cause of death based on medical histories and postmortem CT findings (at least at present), it cannot be said that LLMs can replace radiologists. However, our findings suggest that large-language AI models, in their current state, maybe more effectively utilized as a supportive tool for radiologists in formulating differential cause-of-death diagnoses based on medical histories and postmortem CT findings rather than as a standalone solution for replacing radiologists in the image interpretation process. Version upgrades can lead to improvements in the diagnostic performance of the LLMs [11-13]. Therefore, it is desirable to continue conducting research and evaluations in the future.

## Conclusions

This study demonstrated the usefulness of LLMs in determining the cause of death based on medical histories and postmortem CT findings. Both GPT-4o and Claude 3 Opus showed reasonable accuracy in identifying underlying and immediate causes of death, particularly when provided with detailed input data. While LLMs show promise in assisting with cause-of-death determination for medico-legal purposes, their integration into clinical practice should be approached cautiously, with a clear understanding of their capabilities and limitations. These tools have the potential to enhance the efficiency and accuracy of death investigations, particularly in contexts where specialist resources are limited, but should be used in conjunction with, rather than in place of, expert medical judgment.
